# A 2025 meeting report from the Venice lagoon: International graduate program *RNAmed – Future Leaders in RNA-based Medicine* meets on San Servolo

**DOI:** 10.1261/rna.080962.126

**Published:** 2026-07

**Authors:** Jörg Vogel, Charlotte Kamm, Giorgia Gerolimetto, Pranjal Meel, Alexandre Trubert, Stina Rademacker, Christian Fröschel

**Affiliations:** 1 Helmholtz Institute for RNA-based Infection Research (HIRI), Helmholtz Centre for Infection Research (HZI), Würzburg D-97080, Germany; 2 Medical Faculty, University of Würzburg, Institute of Molecular Infection Biology (IMIB), Würzburg D-97080, Germany; 3 Faculty of Chemistry and Pharmacy, University of Würzburg, Institute for Pharmacy and Food Chemistry, Würzburg D-97074, Germany; 4 Department of Biochemistry, University of Würzburg, Biocenter, Würzburg D-97074, Germany; 5 University Hospital Würzburg, Medical Clinic and Policlinic II, Würzburg D-97080, Germany; 6 Department of Pharmacy, Pharmaceutical Technology and Biopharmacy, Ludwig-Maximilians-University Munich, Munich D-81377, Germany

**Keywords:** graduate training program, RNA-centric education, RNA-based medicine, RNA therapeutics, translational research

## Abstract

*RNAmed – Future Leaders in RNA-based Medicine* is a unique graduate training program in the area of RNA-based medicine. Financed by the Free State of Bavaria, Germany, it is jointly run by several universities and research institutes from Würzburg, Regensburg, and Munich. It aspires to equip doctoral students with a comprehensive idea of RNA therapeutics, spanning fundamental biology, translational research, clinical application, regulation, ethics, and societal implications. This integrative approach is meant to cultivate exceptional qualifications for careers across academia, industry, and policy. The *RNAmed* program held its 2025 annual retreat on San Servolo (Venice, Italy), with a total of 40 doctoral researchers, principal investigators (PIs), and invited guests from academia and pharmaceutical industry attending. The three-day program combined keynotes, student presentations, flash talks, a career panel, and topic-table discussions of advances and challenges across RNA therapeutics to catalyze exchange and collaboration. Presentations highlighted emerging directions for antisense strategies, circular RNAs, delivery technologies, and AI-enabled molecular design, reflecting the program's cross-disciplinary nature. Discussions emphasized shared priorities such as clear experimental standards, robust delivery solutions, and stronger academia–industry ties to accelerate safe, effective RNA medicines. Updates on *RNAmed*’s growth and extended funding underscored its mission to develop talent through integrated scientific and professional training. Overall, the retreat strengthened a network of early-career scientists and mentors committed to advancing RNA-based modalities from concept to clinic.

## INTRODUCTION

RNA-based medicine comprises therapeutic strategies that use ribonucleic acids as target, active agent, or guide to direct molecular interventions (Sparmann and Vogel 2023). Key classes include programmable antisense oligonucleotides (ASOs), small interfering RNAs (siRNAs), or microRNAs for mRNAs inhibition or splicing modulation; synthetic mRNA for transient protein expression and vaccination; and CRISPR–Cas platforms for genome editing in vivo. Foundational advances in backbone and sugar chemistries, lipid nanoparticles (LNP), and receptor-targeted conjugates (e.g., GalNAc) have transformed pharmacokinetics and enabled clinical success, exemplified by rapid COVID-19 mRNA vaccines and liver-targeted siRNAs (Polack et al. 2020; Baden et al. 2021; Adams et al. 2023). Case studies in rare monogenic diseases underscore mechanistic precision and the capacity for modular, rapid design. Major challenges include extrahepatic delivery, endosomal escape, immunostimulation, durability, off-target effects, and scalable, quality-assured manufacturing with fit-for-purpose regulation.

The remarkable functional versatility of RNA provides extensive opportunities for therapeutic innovation. RNA-based medicines are progressing rapidly across multiple disease areas—including infectious diseases, cancer, neurodegeneration, metabolic, and rare genetic disorders—and hold the potential to transform patient care and, in some cases, enable durable cures. However, while RNA-centric approaches show great promise for clinical application, this emerging field is underrepresented in the training of young scientists. To address this gap, the graduate program *RNAmed* – *Future Leaders in RNA-based Medicine* was launched in 2022 to recruit an international cohort of excellent doctoral researchers and provide them with structured, interdisciplinary training that integrates both the scientific and societal dimensions of RNA-based medicine. Funded by the prestigious Elite Network of Bavaria (ENB) framework, *RNAmed* is based in the Free State of Bavaria, where several universities host an active and internationally visible RNA research community. In setting up *RNAmed*, projects and PIs were selected with an eye to extensive networking, mutual support, and potential synergy, especially across the partner laboratories (www.rnamed.de).

**FIGURE 1.**
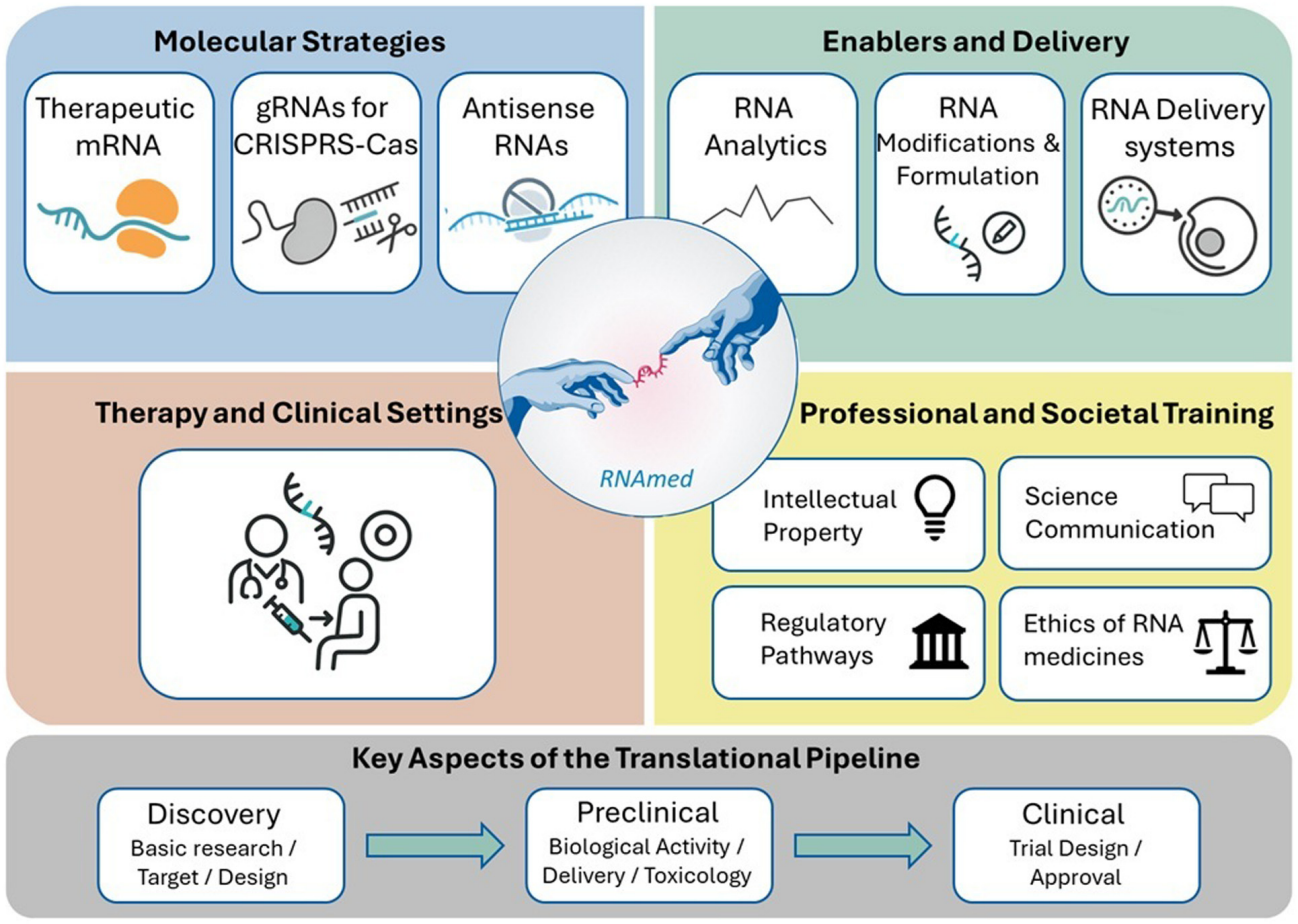
*RNAmed*: From RNA modalities to clinical translation and training. The graduate program *RNAmed* covers the full spectrum of RNA drug/therapy development and seeks to familiarize doctoral students with all aspects of developing an RNA drug or therapy.

Three years into its launch, *RNAmed* has grown to 22 PhD students supervised by 18 different PIs from molecular biology, chemistry, and the pharmaceutical and medical sciences. The program is led by Jörg Vogel, who is based at the University of Würzburg and the Helmholtz Institute for RNA-based Infection Research (HIRI), and runs in close partnership with the University of Regensburg, the Technical University of Munich (TUM), and the Ludwig-Maximilians-Universität München (LMU Munich). Its extracurricular activities offer a high-quality and RNA-specific education that goes beyond what the local graduate schools of the single universities can offer. *RNAmed* has cross-disciplinary and clinically oriented RNA projects and strives to ensure a well-balanced combination of basic biomedical and clinical research. Scientifically, it is focused on three molecular strategies (therapeutic mRNAs, short antisense RNAs, short guide RNAs directing complexes such as CRISPR–Cas systems), strategies for RNA delivery and clinical settings (Fig. 1).

Recognizing that drug development from discovery to approval and rollout is a long, inherently complex process, *RNAmed* extends training beyond the laboratory. Consequently, the *RNAmed* curriculum encompasses molecular principles, RNA analytics, RNA modifications, drug formulation and administration. It further equips students with knowledge of regulatory processes, intellectual property, science communication, and the ethics of RNA medicines. All in all, the mission of *RNAmed* is to prepare talented doctoral researchers for internationally oriented careers in academia or industry, as entrepreneurs, or as policymakers in the expanding field of RNA-based medicine.

### The *RNAmed* Annual Retreat on San Servolo 2025

Since its launch in 2022, *RNAmed* has seen several longer in-person meetings in Germany, for example, the program kickoff retreat and the program evaluation day. However, for the 2025 annual retreat of the program, *RNAmed* went abroad to meet on San Servolo. This Italian island in the Venetian Lagoon transitioned from a medieval Benedictine monastery (9th century) to an 18th-century psychiatric hospital. For over two centuries, San Servolo operated as Venice's principal asylum, until Italy's 1978 psychiatric reform mandated closure and archival preservation. Subsequent restorations reoriented the site toward education and culture. Today, San Servolo hosts Venice International University (of which one of the *RNAmed* universities is a partner) and student residences, maintains a museum with medical records, and serves as a conference and cultural venue.

**FIGURE 2.**
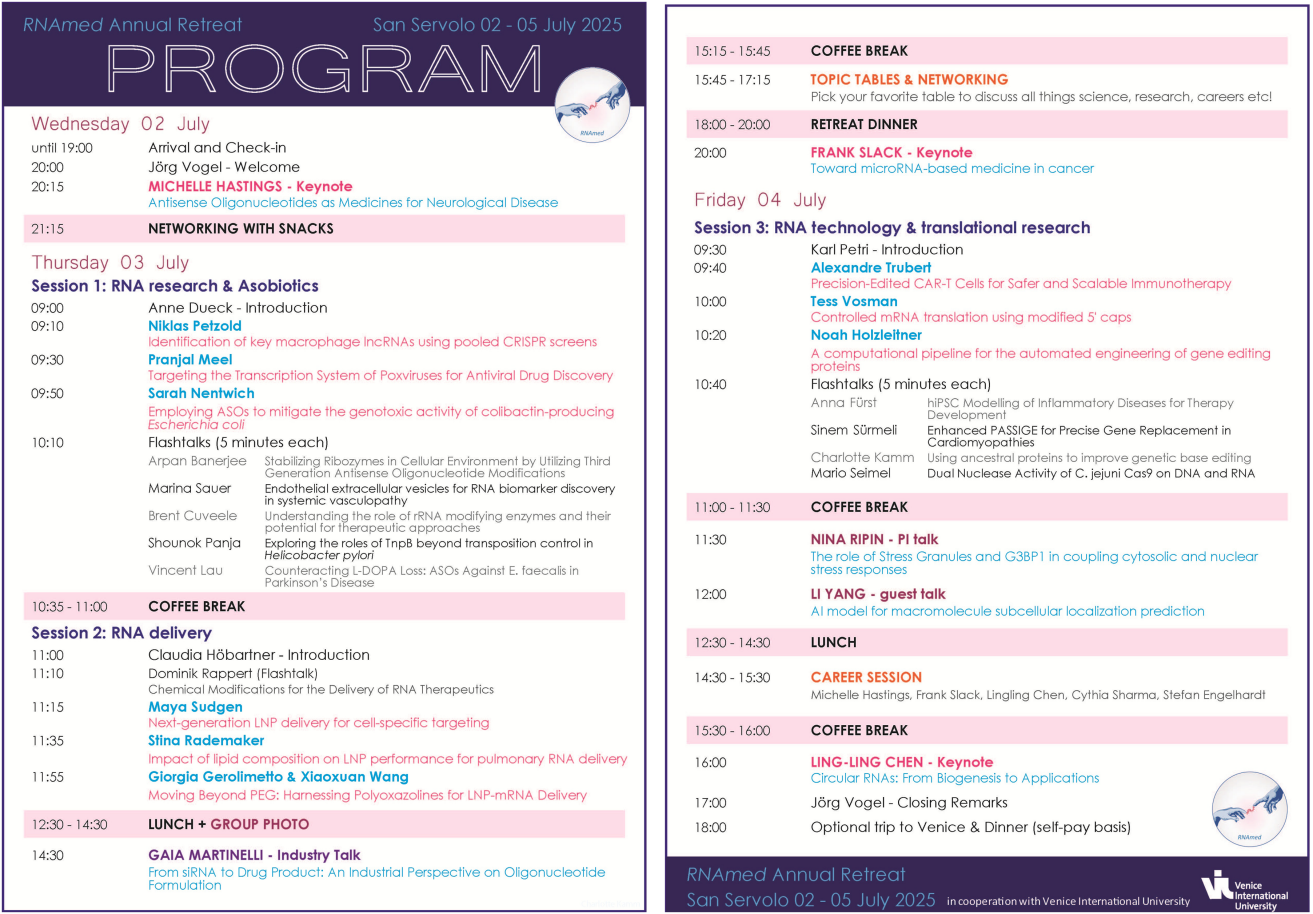
The scientific program of the *RNAmed* Retreat on San Servolo 2025, outline, and design by Charlotte Kamm.

**FIGURE 3.**
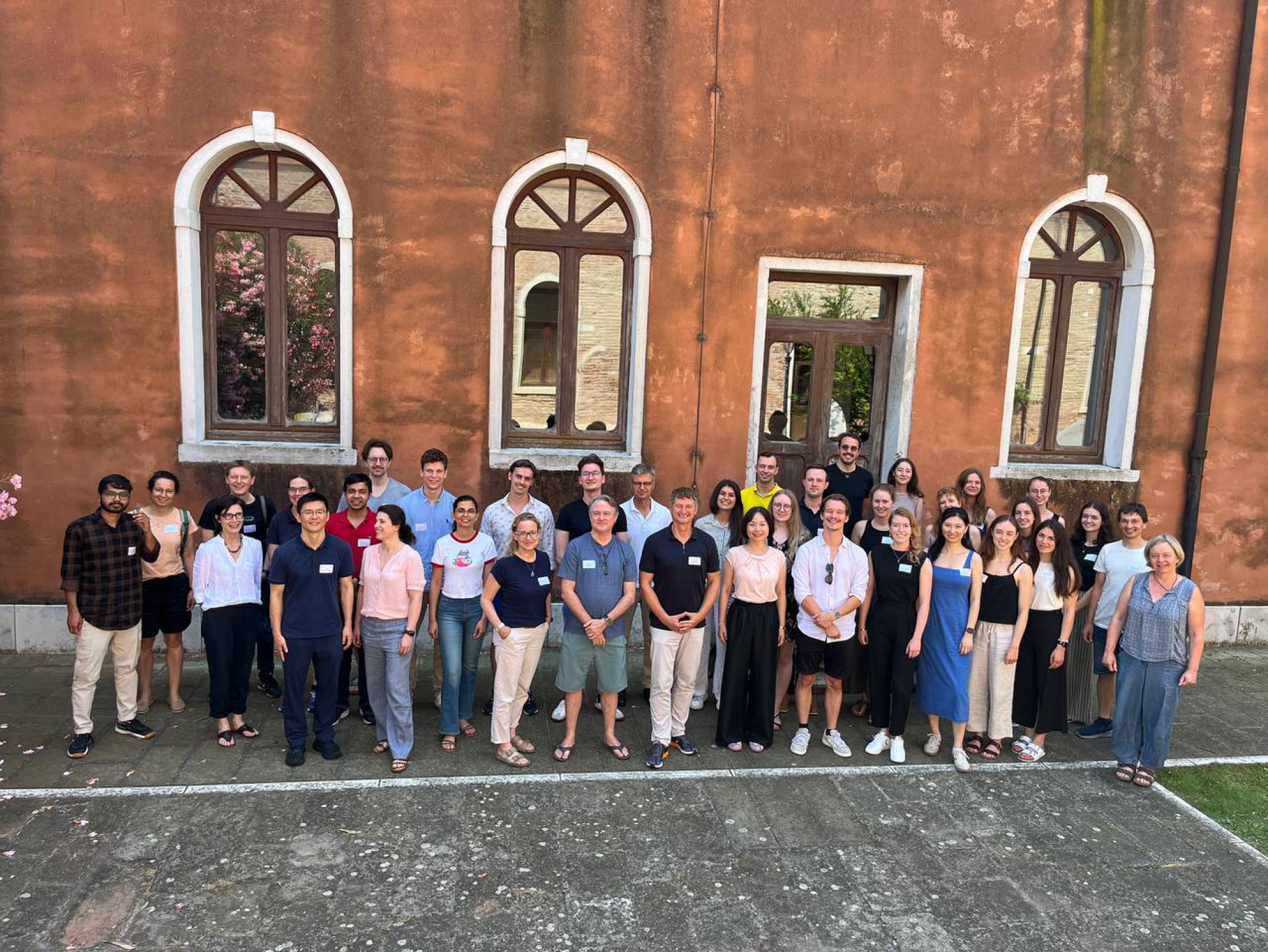
Participants of the *RNAmed* Retreat on San Servolo 2025.

The three-day retreat (Fig. 2), held on July 2–5, 2025, brought together *RNAmed* PIs and PhD students and invited guests from industry and academia. There were more than 40 participants from 11 countries and multiple disciplines, including basic RNA biology, RNA chemistry, clinical research, and bioinformatics (Fig. 3). The agenda had been written by the PhD students and comprised three sessions, each of which featured three 20 min research talks by PhD students, complemented by 5 min flash talks. Each day saw a different keynote lecture by external speakers. The second day included table discussions and a retreat dinner, offering ample opportunities for networking among the students, PIs, and guests. The retreat concluded with a career session that enabled students to engage with and pick the PIs’ brains about career paths in the field of RNA-based medicine.

In his welcome remarks, *RNAmed* spokesperson Jörg Vogel introduced his co-organizers and outlined the structure and objectives of the graduate program. He started off with the good news that the Bavarian ministry had just approved the second funding period of *RNAmed*, from 2027 until 2030 (the present funding ends in November 2026). The continued funding will help to refine the *RNAmed* concept, further strengthening the profile and visibility of the program. The call for applications for the second cohort of doctoral students will go out in spring 2026. He also highlighted potential synergies between *RNAmed* and the new Cluster of Excellence NUCLEATE (Cluster for Nucleic Acid Sciences and Technologies), which will commence in 2026 as a collaboration between the University of Würzburg and the two Munich universities (TUM and LMU). NUCLEATE unites leading researchers, cores, and partners to decode, engineer, and translate nucleic acids into next-generation diagnostics and therapies.

## SCIENTIFIC PROGRAM

### Keynote lectures

Michelle Hastings and Frank Slack are both members of *RNAmed*’s Scientific Advisory Board (SAB). Their presence during the retreat offered excellent opportunities to provide them with a comprehensive overview of the graduate program and to invite them to give keynote speeches. The third keynote lecture was delivered by Ling-Ling Chen.

ASOs are short, synthetic nucleic acid sequences that can be designed to base-pair with a specific sequence in an RNA and modulate its expression. They have become a powerful therapeutic platform used to correct aberrant gene expression, providing medicines for diseases that previously had no treatment options. Michelle Hastings (University of Michigan Medical School, Ann Arbor, USA) described a strategy for treating the rare, pediatric neurodegenerative disease, CLN3 Batten disease, using splice-switching ASOs, which induce exon skipping to partially correct gene expression disrupted by frameshifting mutations. An optimized ASO reduced neurological disease burden in mouse models when delivered to the central nervous system and mitigated retinal dysfunction in a pig model after intravitreal injection (Centa et al. 2020, 2023; Stratton et al. 2025). This candidate is advancing to clinical development. Building on prior results, the team further created an exon-8–skipping ASO targeting rare *CLN3* variant c.569dupG. The lead ASO, Zebronkysen, was identified and, after rigorous safety testing, received FDA approval to treat twins with this variant (https://www.forebatten.org/zebronkysen). To date, they have received five intrathecal doses via lumbar puncture at three-month intervals. Zebronkysen has been well tolerated with no severe drug-related adverse events. Over the first year, there was no disease progression or loss of abilities and improvements in motor function, mood, behavior, and autonomic function, supporting ASO-mediated exon skipping.

**FIGURE 4.**
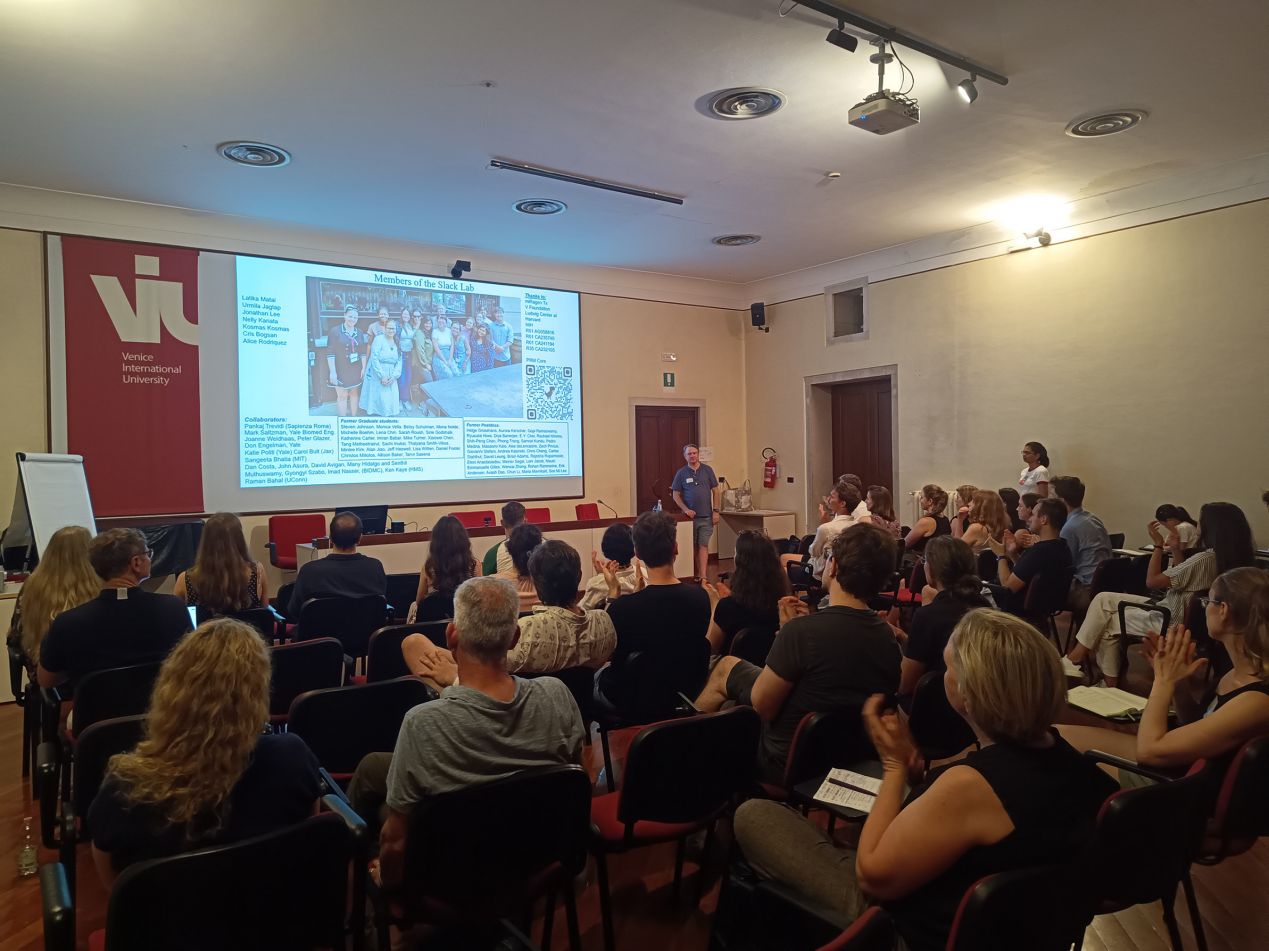
Keynote lecture by Frank Slack (Harvard Medical School).

Frank Slack (Harvard Medical School) outlined in his presentation recent advances in microRNA-based medicine, emphasizing microRNAs as central noncoding regulators of gene expression and cancer pathogenesis (Slack and Chinnaiyan 2019). It surveys their emerging roles as minimally invasive diagnostic biomarkers and as therapeutic agents or targets. This includes strategies involving a miR-21 mimic in preclinical models during liver disease progression (Jagtap et al. 2025), as well as antimiR strategies against the highly expressed oncogenic miRNA-155 in B-cell malignancies (Anastasiadou et al. 2021). Experimental and early clinical data demonstrate that precise modulation of microRNA networks can reverse malignant phenotypes, support personalized theranostic (diagnostic and therapeutic target) approaches, and motivate development of integrated platforms for RNA detection, functional analysis, and targeted delivery (Fig. 4; Pradeep et al. 2023).

Ling-Ling Chen (Shanghai Institute of Biochemistry and Cell Biology, CAS, China) spoke about circular RNAs, highlighting their unique biogenesis, stability, and conformation that enable distinct cellular functions and therapeutic potential. She summarized her laboratory's work on circular RNA metabolism and how RNA conformation controls the low immunogenicity of synthetic RNA circles (Liu et al. 2019, 2022). Chen also presented the development of unmodified ds-cRNA aptamers (circular RNAs with short intramolecular duplexes) that alleviated inflammation in models of psoriasis (Guo et al. 2025), osteoarthritis, and Alzheimer's disease. Finally, she shared the first-ever FDA-approved IND for a circular RNA therapy to treat radiation-induced xerostomia (NCT06714253).

#### Session 1: general RNA research and ASOBIOTICS

In her introduction to the first session of the retreat, session chair Anne Dueck (TUM) presented research on the role of long noncoding RNAs (lncRNAs) in cardiac macrophages, focusing on the lncRNA *Schlafenlnc* (Dueck et al. 2025). This lncRNA is highly expressed in cardiac macrophages and regulates inflammation and fibrotic signaling in the heart. Using single-cell RNA-seq and in vivo experiments, the study revealed functional impacts of *Schlafenlnc* knockout. Protein interaction analyses identified potential partners involved in transcriptional regulation, highlighting *Schlafenlnc* as a key modulator of cardiac immune responses.

The topic was continued by Niklas Petzold, a PhD student in Stefan Engelhardt's group (TUM). He presented his PhD research on the role of the *NIP16* lncRNA in regulating the inflammatory immune response of macrophages. *NIP16*, identified from more than 40 candidates using pooled CRISPR screens, demonstrated a striking effect on a family of C-type lectins, pattern recognition receptors critical for sensing bacterial and fungal pathogens. Loss of *NIP16* led to impaired macrophage activation in response to mycobacterial stimuli. Ongoing work will explore *NIP16*’s function in disease models using knockout mice and assess its therapeutic potential supported by its strong evolutionary conservation in humans.

Pranjal Meel, a PhD student in Utz Fischer's group (University of Würzburg), presented her work on targeting the poxvirus transcription system. Poxviruses are double-stranded DNA viruses, and some (e.g., Mpox) pose significant zoonotic risks. However, they not only just cause diseases but are also used as live vaccines and in oncolytic virotherapies. To mitigate risks from poxviral reservoirs, her structure-based drug design strategy targets the virus-encoded transcription machinery. The virus-encoded multisubunit RNA polymerase (vRNAP) orchestrates expression of virtually all poxviral genes and is central to viral propagation (Broyles 2003). Using complementary approaches including high-throughput screening based on fluorescence cross-correlation spectroscopy (FCCS), resistant-mutant analyses, and in vitro transcription assays, her project aims to identify small molecules that selectively target the poxviral RNA polymerase and inhibit viral gene expression (Grimm et al. 2019), thereby limiting infection and spread.

Sarah Nentwich, from Jörg Vogel's group (University of Würzburg and Helmholtz Institute for RNA-based Infection Research), presented the progress of her PhD research. She is part of the antimicrobial ASO team, who focus on precision targeting of mRNAs in diverse microbes and phages (Ghosh et al. 2024; Gerovac et al. 2025; Vogel et al. 2025). She described an ASO approach to mitigating the genotoxic activity of colibactin-producing *Escherichia coli*. Focusing on rogue commensals, she discussed how ASO-mediated mRNA inhibition in colibactin-producing *E. coli* strains might eventually help to prevent colorectal cancer.

The session ended with several flash talks by other PhD students of the *RNAmed* program.

#### Session 2: RNA delivery

The session opened with a flash talk on chemical modifications for the delivery of RNA therapeutics given by a PhD student from the group of Claudia Höbartner (University of Würzburg), the latter of whom also chaired the session. Next, Maya Sugden from the group of Julian Grünewald (TUM) presented her work on de novo binder (DNB) conjugated LNPs as a promising system for cell receptor targeted delivery. Using DNBs designed with BindCraft (Pacesa et al. 2025), Maya demonstrated preferential delivery of mRNA to K562 cells expressing either *HER2* or *EGFR*, against a panel of other receptors. She provided the audience with an overview of current approaches to active nonhepatic targeting of LNPs, and discussed the benefits of DNBs over antibodies as a cheaper and faster approach for generating novel binding moieties for new targets of interest.

Stina Rademacker, a PhD student in the group of Olivia Merkel (LMU Munich), presented LNP formulations for pulmonary RNA delivery. LNPs represent a highly promising platform for pulmonary RNA delivery (Akinc et al. 2019; Sarode et al. 2024). Her presentation highlighted how variations in LNP composition such as helper and PEG lipids, as well as RNA cargo, affect key properties, like transfection efficiency, endosomal escape, mucus penetration, and protein corona formation. Employing conventional submerged culture and a physiologically relevant air–liquid interface lung model, both the lipid composition and RNA cargo were demonstrated to critically influence LNP performance (Rademacker et al. 2025). These findings provide valuable insights into how lipid compositions shape the efficiency of RNA-based therapies for respiratory diseases.

The session concluded with a double presentation by Giorgia Gerolimetto and Xiaoxuan Wang, both PhD students in Lorenz Meinel's group (University of Würzburg). They presented their work on the formulation design of LNPs for mRNA delivery, focusing on polyoxazoline-based polymeric materials as a replacement for polyethylene glycol (PEG) lipids. The use of alternative stealth polymers, such as polyoxazolines, addresses immunogenicity concerns commonly associated with PEG (Chen et al. 2025), thereby opening new avenues for improved therapeutics (Weber et al. 2025). A rational design of experiments (DoE) approach (Daniel et al. 2022) was taken to characterize the complex design spaces of LNPs, determining key factors to obtain particles with desirable quality attributes.

#### Session 3: RNA technology and translational research

Session chair Karl Petri (University Hospital Würzburg) opened the session by discussing recent advances in the clinical translation of RNA-guided gene editing, focusing on off-target analysis and genomic safety. His team develops high-resolution methods to measure unintended edits and applies these tools to systematically benchmark emerging “CRISPR 2.0” editors, which include base editors and prime editors. The hope is that these new technologies can overcome some of the key limitations of classical CRISPR systems, particularly in the context of engineered immune cells. Petri also outlined strategies for translating these technologies into next-generation CAR-T cell therapies, where enhanced precision, predictable repair outcomes, and robust safety profiling are essential for clinical use (Petri et al. 2025).

A PhD student in Petri's laboratory, Alexandre Trubert (University Hospital Würzburg), continued by presenting the promising results of SLAMF7-directed CAR-T cell therapy in multiple myeloma (Prommersberger et al. 2021), but its broader application is limited by autologous manufacturing and fratricide during production. To overcome these limitations, he develops an improved allogeneic SLAMF7 CAR-T product using mRNA-based base editing (Webber et al. 2019) to disrupt *TCR*, *HLA-I*, *HLA-II*, and *SLAMF7*. The edited cells exhibited strong expansion and potent cytotoxicity, supporting the development of a scalable, off-the-shelf immunotherapy against multiple myeloma. This work shows the potential of base editing as a versatile and efficient tool to generate universal, fratricide-resistant CAR-T cells.

mRNA is an emerging medical modality; however, approaches to control its activity lag behind other biologics. PhD student Tess Vosman from the group of Andrea Rentmeister (LMU Munich) discussed her approach to controlling mRNA translation using 5′ cap analogs that block the binding of the eukaryotic translation initiation factor 4E (eIF4E). In cells, translation of an ectopic mRNA with this 5′ cap analog is blocked until the 5′ cap modification is removed. She presented different types of modifications that can be removed by different triggers, such as light (Klöcker et al. 2022) or a small molecule. She discussed the release mechanisms, synthesis/purification of the mRNA, and in-cell applications.

Noah Holzleitner, a PhD student in the group of Julian Grünewald (TUM), presented his laboratory's work on FORGE, a new pipeline designed to make engineering gene-editing proteins more accessible. He explained that while many recently discovered Cas proteins and recombinases have strong potential, they often require optimization to work efficiently in human cells. Traditional engineering methods can improve performance but are complex and difficult for smaller laboratories to adopt (Krapp et al. 2023; Gilchrist et al. 2024; van Kempen et al. 2024). FORGE predicts potentially improved variants using only an amino acid sequence, allowing researchers to skip heavy computational work. Noah highlighted how his team uses FORGE to enhance two miniature Cas12λ enzymes and a serine recombinase, helping democratize protein engineering.

This session, too, concluded with a number of 5-minute flash talks by several other *RNAmed* PhD students.

#### Guest talks

Next to MSc and doctoral programs, the ENB funds Junior Research Groups at Bavarian universities. Their research shall align with the topic of an active ENB doctoral program and be integrated into the latter. Since April 2025, Nina Ripin (University of Regensburg) has been an associate junior research group leader at *RNAmed*. She investigates the properties, function, and disease relevance of stress-induced ribonucleoprotein (RNP) granules using interdisciplinary approaches. The retreat provided an excellent opportunity for her to get to know the entire *RNAmed* group personally. On San Servolo, she presented her postdoctoral work on stress granules and RNA helicases conducted at CU Boulder (USA). Stress granules are membraneless assemblies enriched in proteins and RNAs that form under cellular stress. She highlighted a perspective of stress granules as RNA aggregates whose composition and stability arise from promiscuous *trans* RNA–RNA interactions (Ripin and Parker 2022). These interactions are regulated by RNA chaperones, proteins that remodel RNA networks and shape granule formation and dissolution (Tauber et al. 2020; Ripin and Parker 2023). By combining biochemical and imaging approaches, her laboratory aims to uncover principles of RNA homeostasis and mechanisms preventing pathological RNA aggregation, with implications for stress response and disease.

Li Yang (Fudan University, Shanghai, China) presented two artificial intelligence (AI) models for macromolecule subcellular localization prediction. First, he showed a machine learning model, called RNAlight, to identify nucleotide k-mers contributing to the subcellular localizations of mRNAs and lncRNAs. Importantly, RNAlight can also be extended to predict the subcellular localizations of other RNAs, including circular RNAs (Yuan et al. 2023). Second, he presented a deep generative model, called deepGPS and a related website (https://bits.fudan.edu.cn/opengps), for protein subcellular localization prediction, which can predict cytoplasmic and nuclear localizations by reporting both textual labels and generative images as outputs (Yuan et al. 2025).

Gaia Martinelli is an industry scientist at Novartis AG specializing in the pharmaceutical development of RNA/oligonucleotide therapeutics, particularly the formulation of siRNA-based drugs into robust drug products. Her expertise spans the path from molecule design to market-ready products, including oligonucleotide stabilization, formulation and excipient strategy, delivery route and device selection, and navigating regulatory requirements for these complex modalities. In her presentation, she nicely complemented the scientific program with an industry perspective. She shared pharmaceutical insights on the development of oligonucleotide therapeutics, outlining the journey from molecule design to delivering a marketable product to patients and highlighting challenges and opportunities in this evolving field. Her talk incorporated insights from a recent review of commercial oligonucleotide drug products (Vinjamuri et al. 2024), addressing strategies for molecular stabilization, formulation considerations such as buffer and excipient selection, administration routes, and device choice, as well as regulatory aspects. The presentation emphasized the complexity of this development process and the factors influencing successful market entry.

#### Topic tables

**FIGURE 5.**
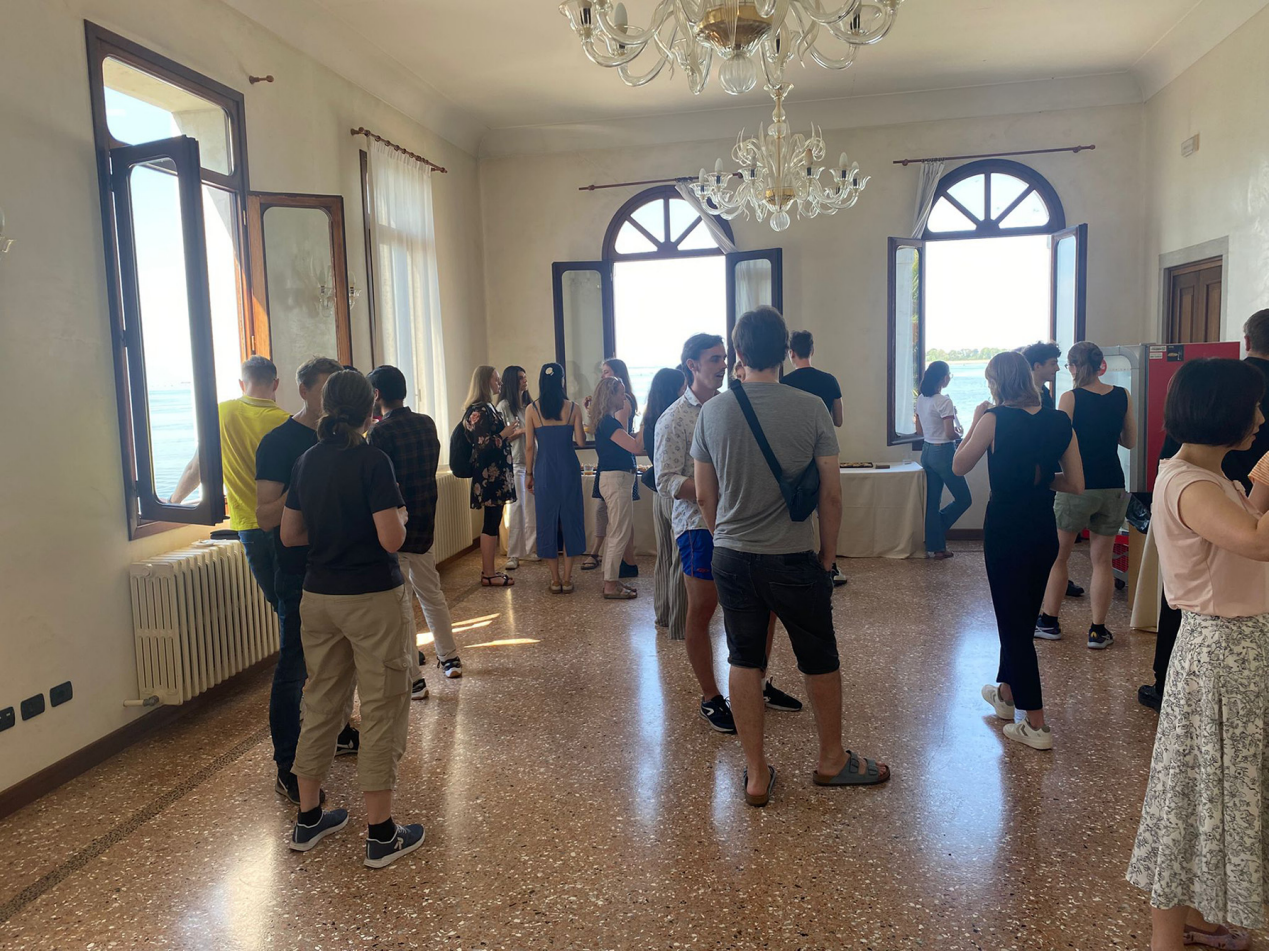
One of the many opportunities for scientific networking during breaks.

**FIGURE 6.**
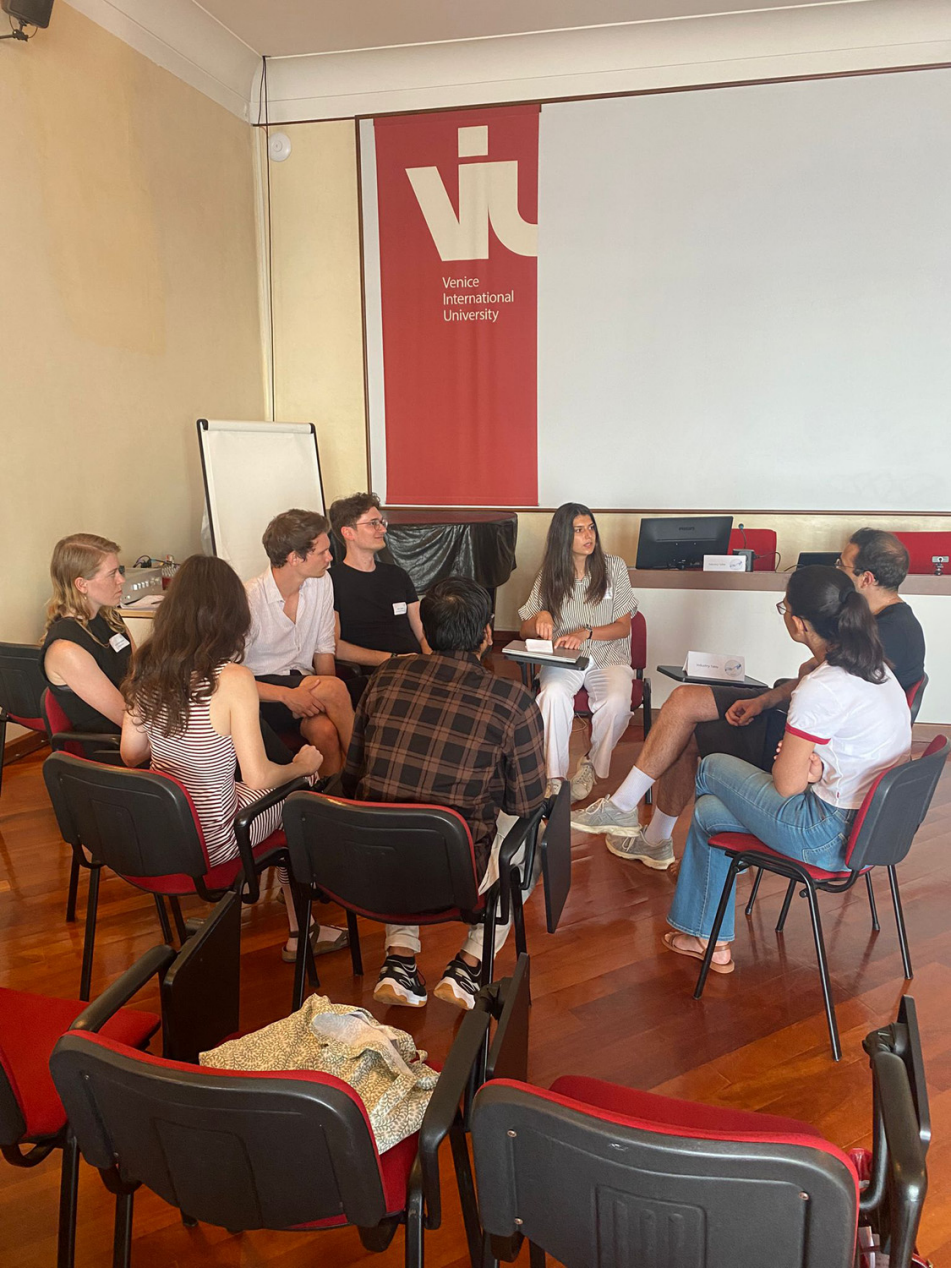
*RNAmed* students discussed industry perspectives at a Topic Table with Gaia Martinelli (center) from Novartis AG.

*RNAmed* thrives on its diversity of scientific topics, fields of interest, and experience levels among its students and PIs. While there is a shared interest in advancing RNA-based medicine, the approaches to achieving this are highly individual. The Topic Tables aimed to capture the various areas of *RNAmed*, ranging from scientific matters to professional issues such as job opportunities in industry and academia. At the same time, it facilitated scientific networking by providing conversation starters and discussion topics. PIs were assigned specific topics and tables to discuss, such as the relationship between academia and industry, the challenges faced by early-career scientists, or CRISPR therapies. Students were then free to move between tables of interest, listening to and contributing to ongoing conversations. This allowed for casual conversation and productive discussion (Figs. 5, 6).

#### Career session

The last day of the retreat featured a career session during which the *RNAmed* students had the opportunity to interact with SAB members Michelle Hastings and Frank Slack, invited speaker Ling-Ling Chen and *RNAmed* PIs Cynthia Sharma (University of Würzburg), Stefan Engelhardt (TUM), and Gunter Meister (University of Regensburg). The session, chaired by *RNAmed* student Charlotte Kamm, provided an open and friendly atmosphere with lively conversations. Although there was no strict agenda, the moderator guided the conversation from the professional aspects of the PIs’ careers, such as significant turning points, to more personal questions, such as how to balance a successful career with family life. One important matter for many students was how to choose the right career path. In particular, a career in academia raised questions about stress levels, responsibilities, and work–life balance. The PIs helped to put these concerns into perspective by sharing how they are navigating the demands of academia in a sustainable way, offering encouraging insights and guidance for students considering this route.

## SUMMARY AND OUTLOOK

**FIGURE 7.**
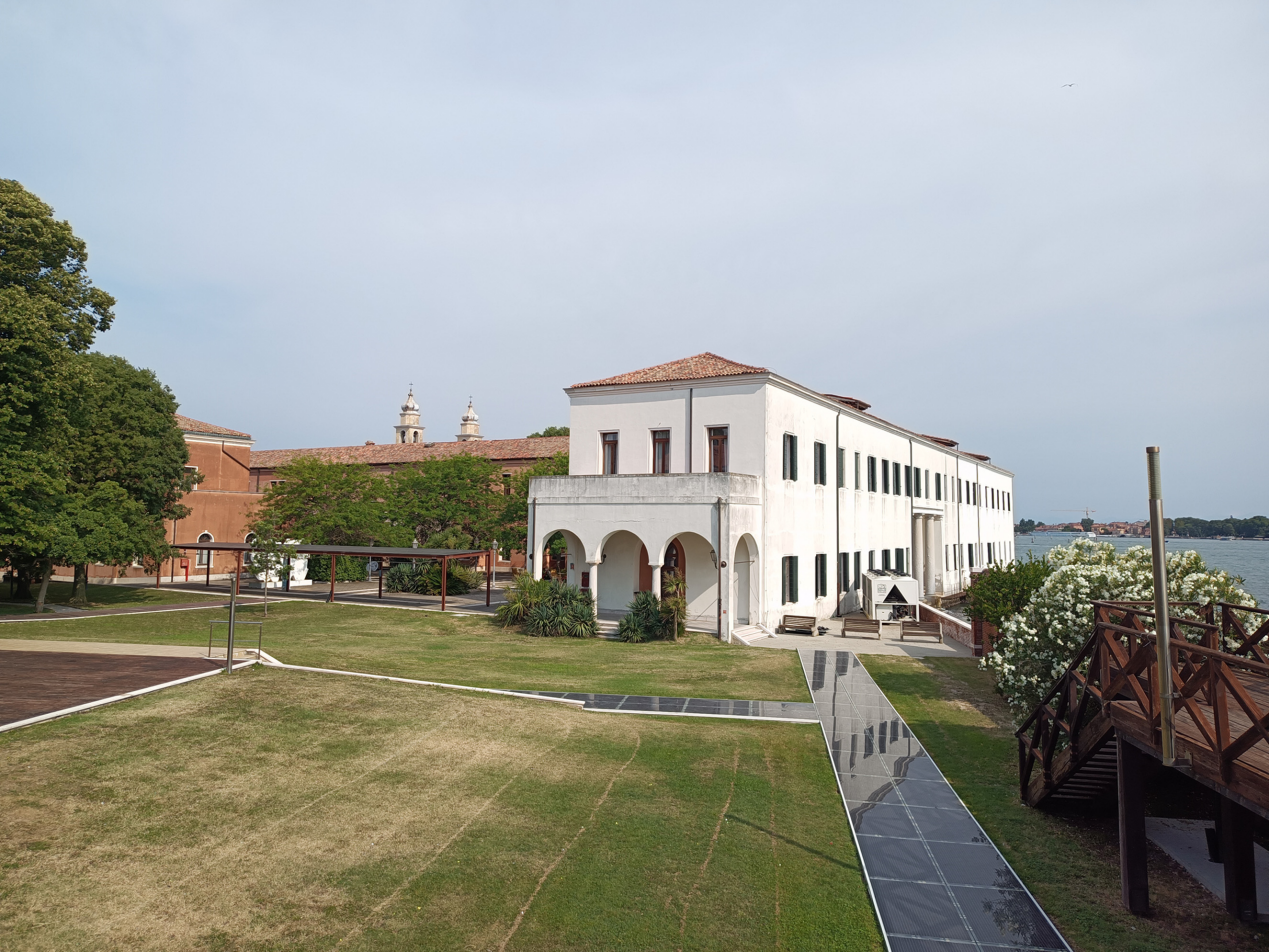
The venue next to monastery buildings on San Servolo.

**FIGURE 8.**
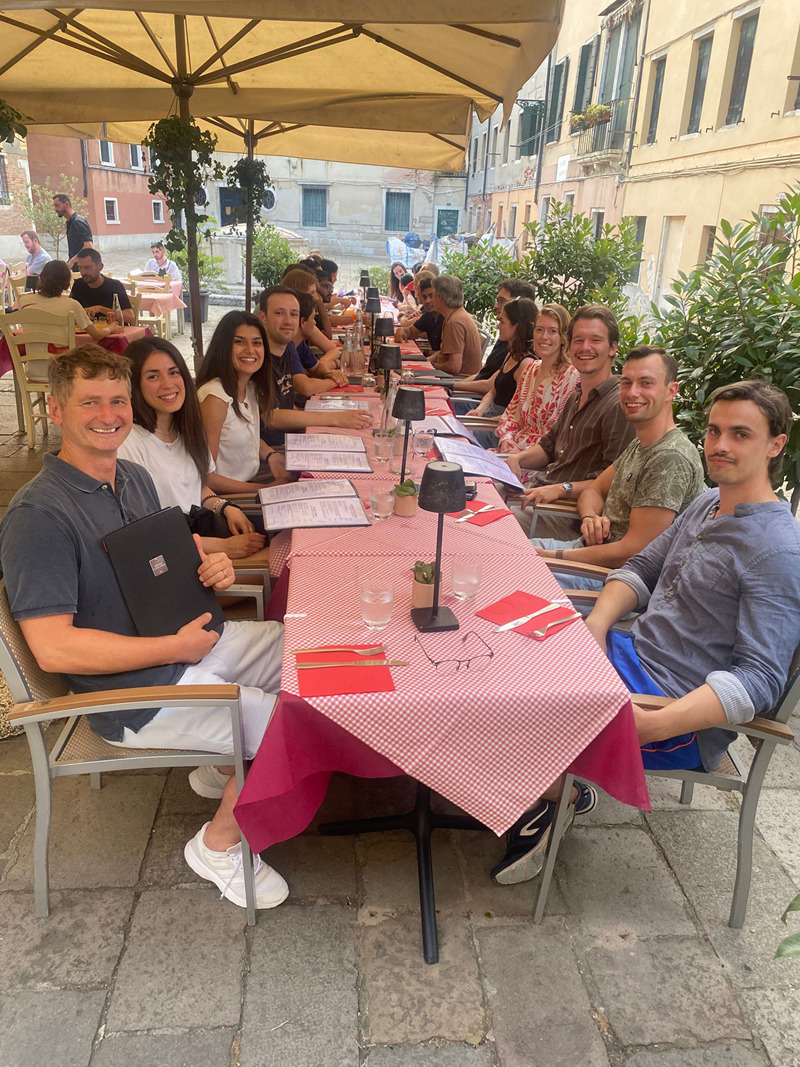
Closing dinner on the Venice mainland.

San Servolo provided a great setting for a scientific retreat (Figs. 7, 8). The remote island location and informal atmosphere allowed the attendees to dive deep into RNA-based medicine, with sustained discussion beyond the formal program. A student-organized meeting featuring predominantly student speakers, it created a relaxed atmosphere, with PhD candidates and PIs interacting at eye level in talks, discussions, and social activities. The combination of plenary sessions, small-group topic tables, and a dedicated career session supported both in-depth scientific feedback and reflection on career paths. Participants particularly valued the opportunity to present their projects in an interdisciplinary environment, receive constructive feedback from peers and PIs, and engage in open conversations about science and careers that are less accessible at larger, more formal conferences.

The retreat was regarded as truly representative of the *RNAmed* PhD program, which combines high-quality scientific training with a strong emphasis on community building and student participation. Students reported that the program enabled them to develop their own perspective on RNA-based medicine while benefiting from expert, collegial supervision and mentorship. They highlighted the well-structured, student-oriented training activities, which have advanced both their research projects and professional development. International students, in particular, emphasized that the program has helped them integrate into the German scientific landscape.

*RNAmed* exemplifies a structured graduate program centered on RNA-focused PhD projects, a cross-campus, cross-disciplinary design, and training beyond the bench (e.g., regulation, intellectual property, ethics, communication). With this profile, *RNAmed* addresses a gap in the current landscape of structured biomedical graduate programs. Placed in Bavaria's vibrant RNA ecosystem, *RNAmed* has the potential to serve as a role model for other research areas. Its graduates will be well positioned to bridge science, regulation, and translation across academia, industry, entrepreneurship, and policy.

International networking is a main pillar of *RNAmed*, leveraging the extensive network of personal contacts of the involved PIs, including partners at RTI Worcester or the Shanghai Institute of Biochemistry and Cell Biology. Neighboring Austria and Switzerland host collaborative research centers highly relevant to *RNAmed*. The program has already built ties with the Austrian SFB (F80) RNA-DECO, a collaborative research center devoted to how chemical RNA modifications influence function, and with the Swiss National Centre of Competence in Research (NCCR) RNA & Disease. The *RNAmed* students and PIs will soon meet their Swiss, Austrian, and French partners at the symposium “Molecular Mechanisms of RNA in Diseases” in St. Moritz, Switzerland, in January 2026. This symposium will promote idea exchange and collaboration between leading scientists and early-career researchers and will serve as the starting point for closer collaboration with neighboring countries.
